# Sigmoidoscopic extent of ulcerative colitis and associated factors in Pakistani population

**DOI:** 10.12669/pjms.38.1.4648

**Published:** 2022

**Authors:** Asim Saleem, Mubashar Zeeshan, Faryal Hazoor, Ghulam Mustafa

**Affiliations:** 1 Dr. Asim Saleem, FCPS (Medicine), Associate Professor of Medicine, Gujranwala Medical College/Teaching Hospital, Gujranwala, Pakistan; 2 Dr. Mubashar Zeeshan, MBBS, FCPS, Medical Officer, Liver Clinic, Jail Road, Lahore, Pakistan; 3 Dr. Faryal Hazoor, MBBS, FCPS, Medical Officer, Liver Clinic, Jail Road, Lahore, Pakistan; 4 Dr. Ghulam Mustafa, PHD, Assistant Professor, Department of Computer Sciences, Bahria University, Lahore, Pakistan

**Keywords:** Ulcerative colitis, Sigmoidoscopic procedure, Retrospective cohort study, SPSS

## Abstract

**Objectives:**

To determine the extent of ulcerative colitis and associated factors in patients who underwent sigmoidoscopy at Liver Center, Jail Road, Lahore, Pakistan.

**Methods:**

In this retrospective cohort study, patients who underwent sigmoidoscopy from July 2013 to July 2020 at Liver Clinic, Jail Road, Lahore, were categorized into two cohorts: who had ulcerative colitis confirmed on histology and who had no ulcerative colitis. Extent and severity of the disease as well as coexisting pathologies were also noted. SPSS version 25 was used. Independent sample T-test was applied to compare quantitative variables like age and weight, and chi-square test to compare qualitative variables with two cohorts. The p-value less than 0.05 was opted as significant. Odd ratio with 95% confidence interval (CI) were also computed for each association.

**Results:**

About 11.55% patients (165 out of 1428) had ulcerative colitis, whose mean age and mean weight were 38.27 ± 14.15 years and 74.08 ± 13.20 Kg respectively. Among ulcerative colitis patients, 18.2% had proctitis, 22.4% had proctosigmoiditis, 27.7% had left-sided colitis, and 31.5% had extensive colitis. May endoscopic severity score was found 0,1,2, and 3 in 12.1%, 23.6%, 31.5%, and 32.7% patients respectively. Ulcerative colitis cohort had significant association with younger age (p<0.01), female gender (p<0.01), non or former smoking (p=0.02) and presentation with bloody diarrhea (p<0.01), and no association with body weight (p=0.311), presence of diabetes mellitus (p=0.311) and family history of IBD (P=0.368).

**Conclusion:**

Endoscopic extent and severity of ulcerative colitis is high in our studied population. Ulcerative colitis is more prevalent in younger age and female gender patients who presented with bloody diarrhea, while the presence of active smoking has negative association with finding the ulcerative colitis. However, presence of family history of IBD, diabetes mellitus and body weight of the patient has no statistical correlation with finding ulcerative colitis during sigmoidoscopic examination in our patients.

## INTRODUCTION

Ulcerative colitis is one type of inflammatory bowel disease that primarily involve mucosa of rectum and colon.^1^ It is idiopathic immune mediated disorder with relapsing and remitting pattern.^2^ It mainly presents with blood in diarrheal stool.^3^ Its incidence is one to two per 100,000 people per year while its prevalence ranges from 0.24 to 7.5 per 100,000 persons in Asian region.^4^ No epidemiological data is available from Pakistan. The disease has multiple complications, where toxic megacolon^5^ is a life-threatening one. Truelove and Witt’s criteria;^6^ tells that how much disease is severe clinically while Mayo score^7^ defines disease severity endoscopically, and Montreal classification,^8^ defines its endoscopic extant. The number of the blood stools more than six, tachycardia, anemia, fever, serum albumin less than 2.8gram per deciliter and ESR more than 30 points severe ulcerative colitis.^9^ Colitis involving beyond splenic flexure, limited till splenic flexure, rectum and sigmoid colon, and only rectum is called extensive colitis, left sided colitis, proctosigmoiditis, and proctitis respectively.

The extent of ulcerative colitis determines prognosis of the disease as well as its clinical consequences. That is why, extensive ulcerative colitis requires vigorous treatment strategies and sometimes surgery even colectomy.^10^,^11^ Likewise, persons suffering extensive colitis have greater chances of developing colorectal cancer in future, whereas those suffering ulcerative proctitis have no threat of this kind.^12^

Little data is available nationally to which.extent and aggression, our people in Pakistan are suffering from this long-lasting disease, and what factors are associated,. This made the author keen to study our patients suffering ulcerative colitis retrospectively. Therefore, the objective of our study was to determine the extent of ulcerative colitis and associated factors in patients who underwent sigmoidoscopy at Liver Center, Jail Road, Lahore, Pakistan.

## METHODS

This retrospective cohort study^13^ was performed at Liver Clinic, Jail Road, Lahore on patients who had sigmoidoscopic examination done from July 2013 to July 2020. Ethical review was obtained on July 15, 2020 (No. Admn 588) from ethical committee of the institution. All the patients who underwent sigmoidoscopy for different lower GI complaints were included and categorized into two cohorts: who had ulcerative colitis and who had no ulcerative colitis. The diagnosis of ulcerative colitis was confirmed by histopathological examination.

All the sigmoidoscopic examinations in our setup were performed till mid-transverse colon, and the disease limited to the rectum was named as ulcerative proctitis while colitis limited to both rectum and sigmoid colon was labelled as proctosigmoiditis. The disease extending till and beyond splenic flexure of colon was named as left sided colitis and extensive colitis respectively. Disease severity was categorized by Mayo endoscopic score. Normal mucosa with intact vascular pattern had score 0, mucosal erythema with decreased vascular pattern had score-1, marked erythema, friability, erosions, and absent vascular pattern had score-2, while score-2 findings plus ulcerations and spontaneous bleeding had score-3. Those four scores were named inactive, mild, moderate, and severe disease respectively.

Based on the indications of sigmoidoscopy, the patients were categorized into two groups: one those who presented with bloody diarrhea and second who presented with complaints other than bloody diarrhea. The patients grouping was also done into having diabetes mellitus and having no diabetes mellitus. During sigmoidoscopy, coexisting colonic pathology like superimposed pseudomembranous colitis, pseudopolyps formation, benign polyps and malignant lesions were also noted.

The age and the weight of the patients were the quantitative variables, while genders, history of diabetes mellitus, and indication for the sigmoidoscopic procedure were the qualitative variables. Statistical Package for Social Science (SPSS), version 25 was used to compute the means and the standard deviations for age and weight of the patients and frequency percentages for the gender, diabetes mellitus history (present/absent) and bloody diarrhea history (present/absent). Independent sample T-test was applied for the comparisons of quantitative variables like age and weight with ulcerative cohorts (ulcerative colitis present / ulcerative colitis absent).

Chi-square test for Independence was used for the comparisons of qualitative variables with ulcerative cohorts (ulcerative colitis present / ulcerative colitis absent). The p-value less than 0.05 was opted as significant. Odd ratio with 95% confidence interval (CI) were also computed for each association.

## RESULTS

Eleven-point fifty five percent patients (n=165) had ulcerative colitis among all 1424 those patients who underwent sigmoidoscopy. The mean age and the mean weight of the patients suffering ulcerative colitis were 38.27 ± 14.15 years and 74.08 ± 13.20 Kg respectively. Among 18.2% patients (n=30), extent of colitis was limited to the rectum only (ulcerative proctitis) and among 22.4% patients (n=37), colitis involved both rectum and sigmoid colon (ulcerative proctosigmoiditis). In 59.4% patients (n=98), ulcerative colitis was seen throughout visualized tract, from rectum till splenic flexure (Table-I).

**Table-I:** Sigmoidoscopic extent of ulcerative colitis disease (n = 165/1424).

	Frequency (Percent)
Ulcerative proctitis	30 (18.2%)
Ulcerative proctosigmoiditis	37 (22.4%)
Left sided ulcerative colitis	46 (27.9%)
Extensive ulcerative colitis	52 (31.5%)

Independent sample T-test showed that mean age of patients suffering ulcerative colitis was significantly lower than that of the patients not suffering ulcerative colitis (38.27 ± 14.15 years vs 44.17 ± 15.56 years) and the association was also statistically significant (p<0.01). On the other hand, weight of the patients was comparable in both groups (74.08 ± 13.20 kgs vs 73.16 ± 13.04 kg) and the statistical association was also insignificant (p=0.311) (Table-II).

**Table-II:** Endoscopic severity of ulcerative colitis (Mayo score) (n = 165/1424).

	Frequency (Percent)
Inactive disease (Score 0)	20 (12.1%)
Mild disease (Score 1)	39 (23.6%)
Moderate disease (Score 2)	52 (31.5%)
Severe disease (Score 3)	54 (32.7%)

**Table-III:** Factors associated with the presence of Ulcerative colitis disease in patients who underwent sigmoidoscopy (n = 165/1424).

Parameters /Categories^*^	Ulcerative colitis		Total	p-value	Odd ratio with 95% Confidence interval

	Yes	No			
Mean Age (Years)	38.27±14.15	44.17±15.56	1424	<0.01	2.032 (1.455 – 2.837)
Mean Weight (Kilogram)	74.08±13.20	73.16±13.04	1424	0.395	1.081 (-1.199 – -3.040)
***Gender***					
Male	96 (9.4%)	930 (90.6%)	1026	<0.01	2.032 (1.455 – 2.837)
Female	69 (17.3%)	329 (82.7%)	398		
***Smoking***					
Active	33 (8.4%)	361 (91.6%)	394	0.02	0.622 (0.417 – 0.928)
Non or former	132 (12.8%)	896 (87.2%)	1030		
***Diabetes Mellitus***					
Yes	53 (13.2%)	347 (86.8%)	400	0.231	0.804 (0.567 – 1.140)
No	112 (10.9%)	912 (89.1%)	1024		
***Family H/O IBD***					
Yes	17 (14.3%)	102 (85.7%)	119	0.368	1.303 (0.758 – 2.239)
No	148 (11.3%)	1157 (88.7%)	1305		
***Indication of sigmoidoscopy***					
Bloody diarrhea	147 (22.4)	511 (77.6%)	658	<0.01	11.954 (7.235 –19.753)
Others	18 (2.3%)	748 (97.7%)	766		

* Independent sample T-test was used for parameter no 1 & 2, & Chi-square test for independence was used for 3, 4 & 5.

Chi-square test for Independence revealed that female gender was suffering ulcerative colitis significantly more than male gender (p<0.01). Seventeen-point three percent females (69 out of 398 patients) had ulcerative colitis while 9.4% males (96 out of 1026 patients) had ulcerative colitis among patients who underwent sigmoidoscopic examination. Similarly, ulcerative colitis was found during sigmoidoscopy significantly more in patients who presented with history of bloody diarrhea as compared to those patients who presented with complaints other than bloody diarrhea (p<0.01). Twenty-two-point four percent patients with bloody diarrhea (147 out of 658) had ulcerative colitis while 2.3% patients with complaints other than bloody diarrhea (18 out of 766) had ulcerative colitis.

**Fig.1:**
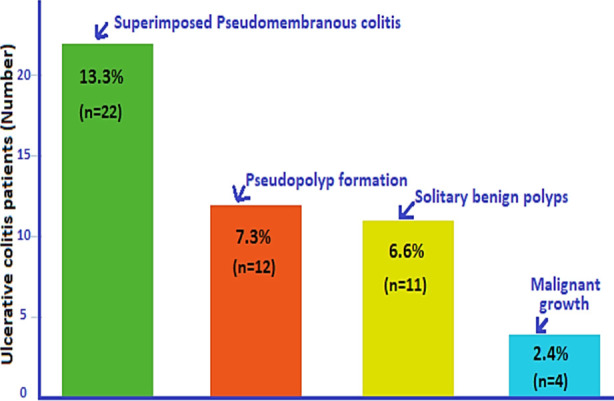
Distribution of coexisting colonic pathology in patients suffering Ulcerative colitis (n=49/165).

However, finding ulcerative colitis during sigmoidoscopy had no significant association with history of diabetes mellitus (p=0.311). Both groups were comparable. 10.4% diabetic patients (52 out of 399) had ulcerative colitis while 11% non-diabetic patients (113 out of 1025) had ulcerative colitis (Table-II). In patients suffering ulcerative colitis, multiple coexisting pathological findings were also noted in the colon. Thirteen point three patients (22 out of 165) had superimposed pseudomembranous colitis, 7.3% (12 out of 165) had pseudopolyps, 6.6% (11 out of 165) had benign polyps, and 2.4% (4 out of 165) had malignant lesions (Fig.1).

## DISCUSSION

Ulcerative colitis is the most common type of inflammatory bowel disease found worldwide. Asian territory has more cases of ulcerative colitis than Crohn’s colitis compared to western countries.^14^ In 2016 Siew C. Ng^15^ studied emerging trends of inflammatory bowel disease (IBD) in Asian region. He found the incidence of ulcerative colitis double than that of Crohn’s colitis (1.0 per 1 X 10^5^ people VS 0.5 per 100,000 1 X 10^5^ people). Ahmed A et al. and colleagues^16^ studied retrospectively patients who underwent sigmoidoscopy in Cork University Hospital, Ireland and found 2% prevalence of ulcerative colitis in a total of 445 Irish people who had sigmoidoscopy done. In our study data, 11.55% (n=165) patients were diagnosed having ulcerative colitis. This may point the burden of the disease in our people, even in comparison to our other neighbor Asian countries as well. Another worryful finding in our study was that Ulcerative colitis was prevalent significantly more at younger age among all those patients who underwent sigmoidoscopic procedure at our setup during last seven years. The mean age of ulcerative colitis patients was 38.27±14.15 years while that of non-colitis patients was 44.17±15.56 years. Internationally, it has been found that there is a rising trend of paediatric IBD.^17^,^18^ Our data favors the same point. Being more cases diagnosed as ulcerative colitis and prevalence of the disease at the younger age is an alarming and apprehensive situation. Local researchers should focus the point to elaborate the things further. In our study, we found ulcerative colitis involving till or beyond splenic flexure in 59.4% patients while ulcerative proctitis in only 18.2%. A large epidemiology study from Asian- Pacific region concluded more prevalence of ulcerative proctitis as compared to extensive colitis among patients suffering ulcerative colitis in our territory (ulcerative proctitis 37% vs extensive colitis 31%).^19^ This is in contrast with our study which points extensive involvement by the disease in our region. Further studies are required to address this issue. John D Betteridge et al^20^ studied 35404 American healthcare providers and found more prevalence of ulcerative colitis in female gender as compared to male gender. On the other hand, a large systematic review of Asian patients suffering ulcerative colitis concluded male prevalence of the disease in our people.^21^ However in our study, we found the ulcerative colitis significantly prevalent in female gender (17.3%) as compared to male gender (9.4%) (p<0.01). There may be a changing trend in gender choice by the disease in our patients with the passage of time. Here, a clue exists for local epidemiologist and may require further workup. Qureshi M et al. and Abbass Z et al. studied clinical presentations of ulcerative colitis patients in Pakistani population.^22^ They found that 90.7%patients presented with mucous diarrhea, where in our study predominant presentation was bloody diarrhea (89% i.e., in 147 out of 165 patients). Our 13.3% ulcerative colitis patients have superimposed pseudomembranous colitis. This ratio is much more than seen in many studies performed worldwide. In a huge USA study^23^ comparing ulcerative colitis patients who presented during sixteen years, the pseudomembranous colitis prevalence was just 3.7%. Undue vigorous antibiotic usage in colitis patients and unhygienicity are usually predispose factors for pseudomembranous colitis.^24^ These are preventable factors and should be addressed to all ulcerative colitis patients who visit our health facilities to reduce the incidence of this coexisting bacterial infection. Lastly, our study also observed no association of finding ulcerative colitis during sigmoidoscopy with history of diabetes mellitus among colitis patients as well as their body weight.

### Limitations of the study:

In order to perform this study, a limited amount of data is collected, and results are generalized over the larger populations. This study is performed with limited resources with the time constraint and limited evidence from the literature.

## CONCLUSION

Left sided and extensive ulcerative colitis is much more common than ulcerative proctitis and proctosigmoiditis in our studied population. Similarly, proportions of patients increase with endoscopic severity Mayo score. This shows high score disease burden in our people. Ulcerative colitis is more prevalent in younger age and female gender patients who presented with bloody diarrhea, while the presence of active smoking has negative association with finding the ulcerative colitis. However, presence of family history of IBD, diabetes mellitus and body weight of the patient has no statistical correlation with finding ulcerative colitis during sigmoidoscopic examination in our patients.

### Authors’ Contribution:

**AS:** Data collection, proof reading and revision.

**MZ:** Literature search and outlining of the paper.

**FH:** Planning and write up.

**GM:** Analysis in SPSS, Consultation, supervision and proof reading, accuracy or integrity of the work.
